# 国产苯达莫司汀联合利妥昔单抗一线治疗惰性B细胞淋巴瘤及老年套细胞淋巴瘤的疗效及安全性——多中心、前瞻性、Ⅱ期临床研究

**DOI:** 10.3760/cma.j.issn.0253-2727.2023.07.004

**Published:** 2023-07

**Authors:** 慧 王, 强 贺, 丹 刘, 秀芝 邓, 骥 马, 琳娜 解, 忠亮 孙, 聪 刘, 荣荣 赵, 可 卢, 晓霞 初, 娜 高, 海臣 魏, 艳花 孙, 玉萍 钟, 立杰 邢, 海燕 张, 颢 张, 文伟 徐, 增军 李

**Affiliations:** 1 山东省肿瘤医院，山东第一医科大学附属肿瘤医院淋巴血液科，济南 250000 Department of Hematology, Shandong Cancer Hospital and Institute, Shandong First Medical University and Shandong Academy of Medical Sciences, Jinan 250000, China; 2 威海市立医院血液科，威海 264200 Department of Hematology, Weihai Municipal Hospital, Weihai 264200, China; 3 济宁市第一人民医院血液科，济宁 272000 Department of Hematology, Jining First People's Hospital, Jining 272000, China; 4 烟台毓璜顶医院血液科，烟台 264000 Department of Hematology, The Affiliated Yantai Yuhuangding Hospital of Qingdao University, Yantai 264000, China; 5 滨州医学院附属医院血液科，滨州 256600 Department of Hematology, Binzhou Medical University Hospital, Binzhou 256600, China; 6 潍坊市人民医院血液科，潍坊 261000 Department of Hematology, Weifang People's Hospital, Weifang 261000, China; 7 青岛市市立医院血液科，青岛 266000 Department of Hematology, Qingdao Municipal Hospital, Qingdao 266000, China; 8 临沂市人民医院血液科，临沂 276000 Department of Hematology, Linyi People's Hospital, Linyi 276000, China; 9 济宁医学院附属医院血液科，济宁 272000 Department of Hematology, Affiliated Hospital of Jining Medical University, Jining 272000, China; 10 山东第一医科大学第一附属医院（山东省千佛山医院）血液科，济南 250000 Department of Hematology, The First Affiliated Hospital of Shandong First Medical University, Jinan 250000, China

**Keywords:** 苯达莫司汀, 利妥昔单抗, 惰性B细胞淋巴瘤, 套细胞淋巴瘤, 治疗, Bendamustine, Rituximab, B cell indolent non-Hodgkin's lymphoma, Mantle cell lymphoma, Treatment

## Abstract

**目的:**

评价国产苯达莫司汀联合利妥昔单抗一线治疗惰性B细胞淋巴瘤（B-iNHL）和老年套细胞淋巴瘤（MCL）的有效性和安全性。

**方法:**

于2020年12月1日至2022年9月10日在山东省内10家三甲医院多中心前瞻性研究国产苯达莫司汀联合利妥昔单抗治疗初诊B-iNHL和老年MCL患者的疗效和安全性，所有患者均已完成至少4个周期诱导治疗。

**结果:**

共入组B-iNHL与年MCL患者72例，中位年龄为55（24～74）岁，美国东部肿瘤协作组体能状态评分（ECOG评分）0～1分者占76.4％，ECOG 2分者占23.6％。滤泡淋巴瘤（FL）占51.4％，边缘区淋巴瘤（MZL）占33.3％，老年MCL占11.1％，不能分类占4.2％。Ann Arbor分期：Ⅲ期占16.7％、Ⅳ期占65.3％。4个周期诱导治疗后，总有效率为98.6％，其中完全缓解（CR）率为83.3％，部分缓解（PR）率为15.3％。仅1例MCL患者治疗中进展，1例FL患者复发。考虑到PET/CT评估与CT评估的差异，即使按照CT评估，其CR率为63.9％。中位随访11（4～22）个月，无进展生存（PFS）率为96.8％，总生存（OS）率为100.0％。主要血液学不良反应包括：3～4级白细胞减少（27.8％，其中8.3％的患者出现粒细胞缺乏伴发热），3～4级淋巴细胞减少（23.6％），3～4级贫血（5.6％）及3～4级血小板减少（4.2％）。非血液学不良反应主要为乏力、恶心/呕吐、皮疹、感染，发生率均低于20.0％。

**结论:**

国产苯达莫司汀联合利妥昔单抗一线治疗B-iNHL和老年MCL患者安全有效。

## Abstract (en)

**Objective:**

This study aimed to assess the efficacy and safety of bendamustine in combination with rituximab（BR regimen）for the treatment of newly diagnosed indolent B-cell non-Hodgkin's lymphoma（B-iNHL）and elderly mantle cell lymphoma（eMCL）.

**Methods:**

From December 1, 2020 to September 10, 2022, a multi-center prospective study was conducted across ten Grade A tertiary hospitals in Shandong Province, China. The BR regimen was administered to evaluate its efficacy and safety in newly diagnosed B-iNHL and eMCL patients, and all completed at least four cycles of induction therapy.

**Results:**

The 72 enrolled patients with B-iNHL or MCL were aged 24–74 years, with a median age of 55 years. Eastern Cooperative Oncology Group（ECOG）performance status scores of 0–1 were observed in 76.4％ of patients, while 23.6％ had scores of 2. Disease distribution included follicular lymphoma（FL）（51.4％）, marginal zone lymphoma（MZL）（33.3％）, eMCL（11.1％）, and the unknown subtype（4.2％）. According to the Ann Arbor staging system, 16.7％ and 65.3％ of patients were diagnosed with stage Ⅲ and stage Ⅳ lymphomas, respectively. Following four cycles of BR induction therapy, the overall response rate was 98.6％, with a complete response（CR）rate of 83.3％ and a partial response（PR）rate of 15.3％. Only one eMCL patient experienced disease progression during treatment, and only one FL patient experienced a relapse. Even when evaluated using CT alone, the CR rate was 63.9％, considering the differences between PET/CT and CT assessments. The median follow-up duration was 11 months（range: 4–22）, with a PFS rate of 96.8％ and an OS rate of 100.0％. The main hematologic adverse reactions included grade 3–4 leukopenia（27.8％, with febrile neutropenia observed in 8.3％ of patients）, grade 3–4 lymphopenia（23.6％）, grade 3–4 anemia（5.6％）, and grade 3–4 thrombocytopenia（4.2％）. The main non-hematologic adverse reactions such as fatigue, nausea/vomiting, rash, and infections occurred in less than 20.0％ of patients.

**Conclusion:**

Within the scope of this clinical trial conducted in China, the BR regimen demonstrated efficacy and safety in treating newly diagnosed B-iNHL and eMCL patients.

惰性B细胞淋巴瘤（B-iNHL）包括滤泡淋巴瘤（FL）、边缘区淋巴瘤（MZL）、淋巴浆细胞淋巴瘤（LPL）及部分分类不明的惰性淋巴瘤或慢性B淋巴细胞增殖性疾病（B-LPDu）等多种类型，具有肿瘤生长缓慢、恶性程度低、自然病程长、无法治愈且复发率高的特点[1]，R-CHOP（利妥昔单抗+环磷酰胺+阿霉素+长春新碱+泼尼松）方案是B-iNHL患者的一线标准治疗[2]。套细胞淋巴瘤（MCL）兼具惰性淋巴瘤及侵袭性淋巴瘤的特征，预后较差[1]。对于年龄小于65岁的MCL患者，接受利妥昔单抗联合含大剂量阿糖胞苷的方案并自体造血干细胞移植巩固目前仍是标准一线治疗[3]。不能耐受大剂量化疗的老年MCL患者，治疗方案与B-iNHL类似。STiL研究[4]和BRIGHT研究[5]–[6]均显示BR（苯达莫司汀+利妥昔单抗）方案的完全缓解（CR）率和无进展生存（PFS）优于传统的R-CHOP/CVP（环磷酰胺+长春新碱+泼尼松）方案。中国临床肿瘤学会（CSCO）指南[7]已经将BR方案列为B-iNHL和老年MCL的一线推荐方案。2019年，苯达莫司汀在国内上市，为了研究含国产苯达莫司汀的BR方案治疗B-iNHL和老年MCL的疗效和安全性，我们发起了本研究。

## 病例与方法

一、病例

本研究为前瞻性、非随机队列研究，自2020年12月1日至2022年9月10日山东省内10家三甲医院共入组72例B-iNHL或老年MCL患者。收集患者的临床资料，包括性别、年龄、美国东部肿瘤协作组体能状态评分（ECOG评分）、疾病分期、有无大包块（≥7 cm）、血清乳酸脱氢酶（LDH）、血β_2_微球蛋白（β_2_-MG）、有无B症状（乏力、盗汗、体重减轻）、结外器官受累情况、有无骨髓浸润、预后评分等。纳入标准：①年龄18～75岁（对于MCL，要求年龄≥65岁，或年龄<65岁但研究者判断存在合并症不适合应用含大剂量阿糖胞苷方案治疗）；②所有患者均在治疗前进行组织活检，病理诊断均符合WHO 2016年淋巴组织肿瘤分类标准[1]；③所有患者既往均未接受过治疗。排除标准包括：①3b级FL；②淋巴浆细胞淋巴瘤/华氏巨球蛋白血症；③在BR方案基础上联合其他化疗药物或靶向药物。不同类型淋巴瘤的治疗指征参考《中国恶性淋巴瘤诊疗规范（2015年版）》[8]。本研究经山东省肿瘤医院伦理审查委员会批准（批件号：SDZLEC2020-176-02），所有患者入组前均签署知情同意书。

二、治疗方案与疗效评估

诱导治疗：BR方案（利妥昔单抗375 mg/m^2^，第0天；国产苯达莫司汀90 mg·m^−2^·d^−1^，第1～2天），28 d为1个疗程，最多应用6个疗程。如果患者出现2次3级血液学不良反应或1次4级血液学不良反应[9]，苯达莫司汀需要下调四分之一剂量，出现3～4级非血液学不良反应也需要下调四分之一剂量。苯达莫司汀减量2次后，如果4周内血液学参数恢复（未发生血液学不良反应或血液学不良反应≤2级）且PLT≥30×10^9^/L，可继续维持减量2次后剂量治疗，否则终止研究。维持治疗：化疗结束后评价疗效，对于达到部分缓解（PR）及以上患者，按照患者意愿进入来那度胺维持治疗组或观察组。来那度胺维持剂量为10 mg/d d1～21，28 d为1个疗程，同时应用阿司匹林75～100 mg/d口服预防静脉血栓。患者完成2、4、6个疗程BR方案治疗后，应用CT或MRI或PET/CT进行疗效评估。疗效评估标准参照2014年Lugano国际工作组标准[10]–[11]。

三、随访

通过查阅患者住院或门诊病历随访及电话随访。末次随访时间为2022年11月10日。总生存（OS）时间定义为自患者入组该临床试验至因任何原因死亡或末次随访时间。PFS时间定义为自患者入组该临床试验至疾病发生进展/复发、死亡或末次随访时间。

四、统计学处理

应用SPSS 26.0和GraphPad Prism 6软件进行统计学分析。计数资料用例数（百分比）表示，计量资料用中位数（范围）表示。采用Kaplan-Meier进行生存分析。双侧*P*<0.05为差异有统计学意义。

## 结果

一、一般资料

72例患者中男33例，女39例，中位年龄55（24～74）岁，年龄大于60岁者30例（41.7％）。ECOG评分，76.4％的患者为0～1分，23.6％的患者为2分。72例患者中，FL 37例（51.4％），MZL 24例（33.3％），老年MCL 8例（11.1％），其他不能分类3例（4.2％）。按照Ann Arbor分期，大多数患者处于Ⅲ期（16.7％）和Ⅳ期（65.3％）。该研究中肿瘤负荷较大的患者占比较高，其中有大包块（≥7 cm）的患者45例（62.5％）。有B症状（发热、盗汗、体重减轻）的患者25例（34.7％），结外器官受累≥2个者25例（34.7％），骨髓浸润者31例（43.1％）。诱导治疗前血细胞减少的患者22例（30.6％）。预后评分高危的患者在50％以上，FL（FLIPI评分≥3分）25例（62.5％），MZL（IPI评分≥2分）13例（59.1％），MCL（MIPI评分≥6分）4例（57.1％）。LDH水平升高者39例（54.2％），血β_2_-MG升高者38例（52.8％）。所有患者均具有系统治疗的指征。

二、疗效及生存

所有患者均完成了至少4个周期的BR诱导治疗。其中60例（83.3％）达CR，11例（15.3％）达PR，客观缓解率（ORR）为98.6％。FL和MZL的CR率分别为89.2％和87.5％，PR率分别为10.8％和12.5％；MCL的CR率为62.5％，PR率为25.0％。仅有1例MCL患者在BR治疗中进展，另有1例FL患者在治疗结束1个月后复发。BR诱导治疗4个周期后，用PET/CT评估疗效的患者为52例（72.2％），CT或MRI评估疗效的患者20例（27.8％）。考虑到PET/CT评估与CT或MRI评估的差异，经PET/CT判定为CR的45例患者中，有13例残存直径>1 cm的淋巴结，将之调整为PR后，矫正后按照CT评估的CR率为63.9％。

中位随访11（4～22）个月。所有患者的PFS率为96.9％，OS率为100.0％。亚组分析显示，FL的PFS率为96.8％，MZL的PFS率为100.0％，MCL的PFS率为87.5％。

三、不良反应

入组的72例患者中，11.1％的患者进行了剂量调整。主要血液学不良反应包括：3～4级白细胞减少（27.8％，其中8.3％的患者出现中性粒细胞减少伴发热），3～4级淋巴细胞减少（23.6％），3～4级贫血（5.6％），3～4级血小板减少（4.2％）。所有级别的非血液学不良反应，如乏力（16.7％）、恶心（18.1％）、呕吐（13.9％）、便秘（13.9％）、腹泻（9.7％）、皮疹（19.4％）、瘙痒（18.1％）、肺部感染（12.5％）、带状疱疹（2.8％）等发生率均低于20％，且多为1～2级。经过对症支持治疗后均得到改善。

## 讨论

B-iNHL是临床常见的淋巴瘤类型，疾病进展缓慢但不能治愈。早期患者可局部治疗或观察，Ⅲ/Ⅳ期患者需要有治疗指征才进行系统治疗[12]–[14]。MCL是较少见的侵袭性B细胞淋巴瘤，但也兼具惰性淋巴瘤的特点，常伴骨髓及胃肠道侵犯。治疗上，对于年龄>65岁或一般状况较差、不适合大剂量阿糖胞苷和自体造血干细胞移植的患者，一线治疗方案与B-iNHL治疗类似[2]。STiL研究[4]和BRIGHT研究[5]–[6]显示BR方案优于R-CHOP/CVP方案，但国内目前没有大系列相关报道。

本研究纳入72例初治的B-iNHL患者，其中以FL（37例）和MZL（24例）为主。入组患者主要为Ⅲ/Ⅳ期（82.0％），结外器官受累的患者占比66.7％，其中骨髓浸润的患者占比43.1％，该比例高于国内其他中心报道的数据[15]，考虑除患者自身特点外，也可能与本中心治疗前分期时应用全身PET/CT（83.3％）且97.2％患者治疗前均行骨髓穿刺及活检检查有关，提高了检出率。

本队列治疗4个疗程后，CR率为83.3％，PR率为15.3％，ORR为98.6％。截至随访终点，仅1例MCL患者治疗中进展，1例FL患者复发，中位随访11个月，PFS率为96.9％，OS率为100.0％。CR率明显高于STiL[4]和BRIGHT[5]–[6]研究（分别40％和31％）。STiL研究和BRIGHT研究中，患者治疗后的缓解情况主要通过增强CT进行评估。我们的研究，72.2％的患者是用PET/CT评估疗效的，CT或MRI评估疗效的患者仅27.8％。其中经PET/CT判定为CR的45例患者中，13例（28.9％）有直径>1 cm的残留淋巴结，如将其判断为PR，则矫正后按照CT评估疗效，CR率为63.9％，仍远高于STiL和BRIGHT研究。这类患者的预后我们将重点关注。GALLIUM研究[16]结果提示对于FL，经过免疫化疗后需要疗效评估的患者，PET/CT比增强CT更准确。另外，2021年发表在JCO上的一篇研究[17]报道了BR一线治疗B-iNHL和MCL的真实世界研究数据，ORR为94.5％，CR率为77.1％，PR率为17.4％，也明显优于STiL研究和BRIGHT研究的结果，而与我们的研究疗效近似。

本研究结果显示，一线应用BR方案产生的不良反应可耐受，其血液学不良反应主要包括中性粒细胞减少、淋巴细胞减少、血小板下降和贫血。非血液学不良反应主要为乏力、恶心/呕吐、皮疹、感染，但所有级别的非血液学不良反应发生率均低于20％，经过对症支持治疗后均得到改善。不良反应发生率与国外报道相似[4]–[6]。本研究有11例年龄≥70岁的患者，其中7例患者出现血液学不良反应，5例出现3～4级中性粒细胞减少，1例发生肺部感染，经过对症支持治疗后血液学参数恢复正常，肺部感染得以控制，无治疗相关的死亡出现。

综上所述，B-iNHL和老年MCL患者一线应用含国产苯达莫司汀的BR方案疗效较好，CR率高于国外十年前临床试验的报道，与近期真实世界研究数据接近，不良反应可耐受。本研究是多中心前瞻性Ⅱ期研究，样本量较小，随访时间较短，对长期生存情况需要进一步随访。
